# Enhanced Memory for Scenes Presented at Behaviorally Relevant Points in Time

**DOI:** 10.1371/journal.pbio.1000337

**Published:** 2010-03-16

**Authors:** Jeffrey Y. Lin, Amanda D. Pype, Scott O. Murray, Geoffrey M. Boynton

**Affiliations:** Department of Psychology, University of Washington, Seattle, Washington, United States of America; Bremen University, Germany

## Abstract

The ability to remember a briefly presented scene depends on a number of factors, such as its saliency, novelty, degree of threat, or behavioral relevance to a task. Here, however, we show that the encoding of a scene into memory may depend not only on *what* the scene contains but also *when* it occurs. Participants performed an attentionally demanding target detection task at fixation while also viewing a rapid sequence of full-field photographs of urban and natural scenes. Participants were then tested on whether they recognized a specific scene from the previous sequence. We found that scenes were recognized reliably only when presented concurrently with a target at fixation. This is evidence of a mechanism where traces of a visual scene are automatically encoded into memory at behaviorally relevant points in time regardless of the spatial focus of attention.

## Abstract 2

What determines whether a scene is remembered or forgotten? Our results show how visual scenes are encoded into memory at behaviorally relevant points in time.

## Author Summary

What determines whether a visual scene is remembered or forgotten? The ability to remember a briefly presented scene depends on a number of factors, such as its saliency, novelty, degree of threat, or relevance to a behavioral outcome. Generally, attention is thought to be key, in that you can only remember the part of a visual scene you were paying attention to at any given moment. Here, we show that memory for visual scenes may not depend on your attention or *what* a scene contains, but *when* the scene is presented. In this study, attention to one task enhances recognition performance for scenes in a second task only in situations when the first task has behavioral relevance. Our results suggest a mechanism where traces of a visual scene are automatically encoded into memory, even though the scene is not the spatial focus of attention.

## Introduction

Photographs of urban and natural scenes can be perceived and understood very quickly. However, to form a memory of a scene requires substantially more processing time. The dissociation between scene perception and memory has been shown by using rapid serial visual presentation (RSVP) to present a series of images and measuring detection and recognition performance while manipulating exposure duration [1]. These studies have shown that scene understanding requires 100 ms or less while memory formation requires at least an additional 300 ms of processing [1]–[5]. The amount of time required for memory formation is dependent on a number of factors; for example, it may take longer to form a memory if images from the set being remembered are highly confusable and similar [4].

There are a number of factors that can increase the memorability of a scene. For example, any feature that increases its “distinctiveness” or novelty—from low-level image features (e.g., a low contrast foggy scene among high contrast daylight scenes) to high-level semantic information—can lead to enhanced scene memory [6],[7]. Novelty is often believed to transiently increase attention, which leads to enhanced memory—a contention supported by experiments suggesting that spatial attention is necessary for a visual item to be encoded into memory [8]–[11]. In addition, the processing of novel events is known to rely on unique neural processing [12]–[17].

Although particularly salient or distinctive information in a scene enhances scene memory, we hypothesized that scene memory would also be enhanced at specific moments in time. A clear example is “flashbulb memory,” where details of the context in which people experience shocking news are stored into long-term memory such as where they were, what they were doing, and with whom they were [18]. This suggests the hypothesis that there may be a mechanism in which unattended (but not necessarily physically salient, novel, or threatening) information is implicitly encoded at behaviorally relevant points in time. We explored this hypothesis by testing participants' ability to recognize a particular scene as a member of a sequence of rapidly presented scenes while performing a demanding detection task at fixation. We found that recognition memory was enhanced for test scenes presented concurrently with an unrelated target at fixation. This is evidence of a mechanism where traces of a visual scene are automatically encoded into memory at behaviorally relevant points in time—operationally defined as a point of time that is important for the future execution or completion of an auditory or visual task—regardless of the spatial focus of attention.

## Results

### Experiment 1

**Figure 1:**
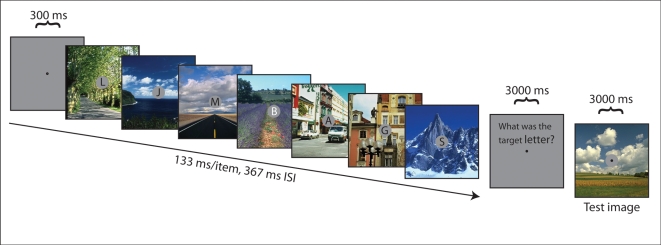
Typical display sequence. Trials were initiated by participants by pressing any letter keys on the keyboard. In Experiment 1, letters at fixation were removed from the displays and participants were instructed to memorize a series of 16 scenes, then to recall whether they recognized a test scene from the RSVP stream (133 ms/item, 367 ms ISI). In Experiment 2, participants were instructed to type the letter key corresponding to the identity of the white target letter for the current trial, then were presented with a test scene and had to recall whether they recognized the scene from the RSVP stream. In Experiment 3, the RSVP letter task was replaced with an auditory task. In Experiment 4, participants received the exact same displays as Experiment 2; however, they were instructed to ignore the letters at fixation and only perform the scene recognition memory task. Prior to testing in every experiment, participants performed a practice block of 24 trials. Each participant was tested for a total of 240 trials, in 10 blocks of 24 trials. Blocks were separated by brief breaks.

**Figure 2:**
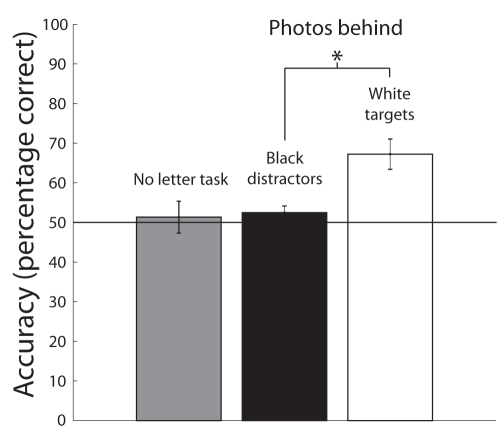
Mean recognition task accuracy for Experiments 1 and 2. Gray bar: results from Experiment 1 where there was no central fixation task. Recognition accuracy was not significantly different from chance performance, 51.32%±4.03%, *t*(11) = 0.3079, *p* = 0.7639. Black bar: results for test scenes presented behind black distractor letters in the target detection task in Experiment 2. Recognition accuracy was again not significantly different from chance performance, 52.49%±1.66%, *t* = 0.5951, *p* = 0.5638. White bar: results for scenes presented concurrently with white target letters in the target detection task in Experiment 2. Recognition accuracy was 67.21%±3.82%. A paired-samples *t* test revealed a significant difference between recognition task accuracy for test scenes that had been previously presented with black distractor letters versus white target letters, suggesting that scenes presented concurrently with white target letters were remembered better, *t*(10) = 2.746, *p* = 0.021. Error bars represent s.e.m. (*p<0.05).

We adapted a standard RSVP task [19] into an RSVP recognition memory task similar to other paradigms used to measure recognition memory for scenes [1],[20]–[22]. In Experiment 1, after being familiarized with a large set of photographs of natural and urban scenes, participants viewed a sequence of 16 scenes presented in an RSVP. Each sequence was then followed by a single test scene in which participants were asked whether they recognized the test scene from the previous RSVP sequence. A typical display sequence is shown in Figure 1. Baseline or chance performance on this task was 50%. Results for the scene recognition task are shown in the grey bar in Figure 2. A *t* test showed that participants performed no better than chance, 51.32%±4.03%, *t*(11) = 0.3079, *p* = 0.7639. Here, participants were unable to recognize whether or not a specific test scene had just appeared in the prior sequence, suggesting a previously unknown difficulty in recognizing a familiar and meaningful scene from short-term memory.

### Experiment 2

In Experiment 2, the same set of scenes was presented, but attention was directed to a demanding task at fixation where the goal was to identify a white target letter among a stream of black distractor letters. As in Experiment 1, one scene was presented immediately after each sequence for the recognition test. Mean performance on the letter identification task in Experiment 2 was 95.22%±1.09%, suggesting that participants were complying with instructions to focus their attentional resources on the fixation task. Results for the scene recognition task in Experiment 2 are shown in the white and black bars in Figure 2. The black bar shows recognition performance for scenes presented during distractor frames (black letters). For scenes presented behind black, non-target letters, performance remained at chance—52.49%±1.66%, *t* = 0.5951, *p* = 0.5638.

Surprisingly, scene recognition was significantly greater than chance for test scenes presented concurrently with white target letters (white bar in Figure 2, 67.21%±3.82%). A paired-samples *t* test reveals a significant difference between recognition task accuracy for test scenes that had previously been presented with black distractor letters versus white target letters, suggesting that scenes presented concurrently with white target letters were remembered better, *t*(10) = 2.746, *p* = 0.021. An additional remarkable feature of Experiment 2 was that participants claimed to have no awareness of their enhanced performance. In debriefing after Experiment 2, all participants claimed that they could not perform the scene recognition task despite performing near 70% on target-present test scenes.

### Experiment 3

We next explored whether this improved performance for scene recognition at the time of target detection was specific to detecting visual targets. Participants performed an auditory target detection task while viewing sequences of scenes as in Experiments 1 and 2. Displays and timing parameters were identical to Experiment 2 except that the alphabetical letters were removed from the scenes and replaced with a fixation marker. With every scene, a baseline auditory tone was presented and a unique tone was designated as the target. Mean performance on the auditory detection task was 90.15%±8.19%, which suggests that participants were complying with instructions to focus their attentional resources on the auditory task.

**Figure 3:**
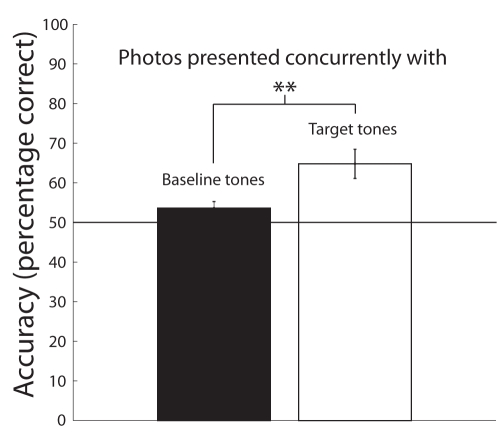
Mean recognition task accuracy for Experiment 3. Photos presented with baseline tones represent trials where the tested scene matched a scene that was presented concurrently with baselines tones in the RSVP stream. Conversely, photos presented concurrently with target tones represent trials where the tested scene matched the scene that was presented with the unique tone in the RSVP stream. In the dual-task condition, recognition accuracy for photos presented concurrently with baseline tones was 53.59%±1.65%, while recognition accuracy for photos presented concurrently with target tones was 64.78%±3.69%. A *t* test reveals that recognition accuracy for photos presented concurrently with baseline tones was not significantly higher than chance levels, *t*(10) = 0.7290, *p* = 0.4827. A paired-samples *t* test revealed a significant difference between recognition task accuracy for test scenes that had been presented concurrently with baseline tones versus target tones, suggesting that scenes presented concurrently with target tones were better encoded into memory, *t*(10) = 3.573, *p* = 0.005. Error bars represent s.e.m. (**p<0.01).

Scene recognition accuracy for Experiment 3 is presented in Figure 3. Similar to Experiment 2, participants performed near chance levels for scenes presented concurrently with distractor tones, 53.59%±1.65%, *t*(10) = 0.7290, *p* = 0.4827. However, performance for scenes presented concurrently with target tones were more accurately encoded into memory, 64.78%±3.69%, *t*(10) = 3.573, *p* = 0.005. This shows that enhanced scene encoding occurs for targets detected across modalities, suggesting that the concept of “behavioral relevance in time” is independent of modality.

### Experiment 4

In both Experiments 2 and 3, the attended targets were perceptually novel compared to distractor stimuli. Thus, enhanced encoding of scenes during target presentation may be simply due to the physical novelty of the stimuli and not due to performing the detection task. To test this, we used stimuli identical to Experiment 2 including the letter stream at fixation, but participants were instructed to ignore the letters and only perform the scene recognition memory task.

**Figure 4:**
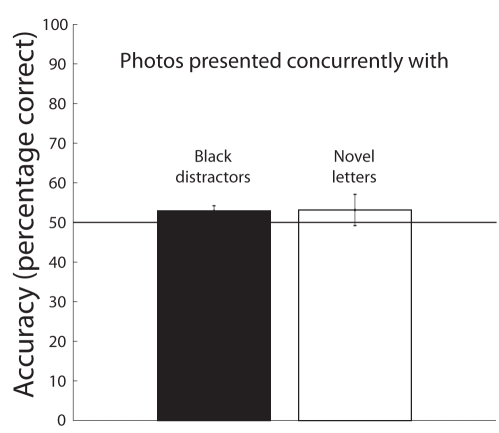
Mean recognition task accuracy for Experiment 4. Photos behind black distractors represent trials where the tested scene matched one of the scenes presented concurrently with a black letter during the RSVP stream. Conversely, photos behind white targets represent trials where the tested scene matched the scene presented concurrently with the white target letter during the RSVP. Displays were identical to Experiment 2; however, participants were instructed to direct their attention to the letters at fixation but only perform the recognition memory task. Given that the white letter serves as a perceptually novel event, one might expect enhanced performance for scenes presented concurrently with the novel event. However, recognition performance was at chance for both test scenes presented concurrently with black distractor letters and with novel white letters, *t*(14) = 0.6798, *p* = 0.5077, and *t*(14) = 0.8373, *p* = 0.4165, respectively. A paired-samples *t* test revealed no significant differences for test scenes presented concurrently with black letters (52.89%±1.33%) and novel white letters (53.13%±3.96%), *t*(14) = 0.1494, *p* = 0.8834, suggesting that the enhanced performance in prior experiments was not simply due to the perceptual novelty of the physical stimulus.

Given that the white letter serves as a perceptually novel event, one might expect enhanced performance for scenes presented concurrently with the novel event. However, recognition performance (shown in Figure 4) was at chance for both test scenes presented concurrently with black distractor letters and with novel white letters, *t*(14) = 0.6798, *p* = 0.5077, and *t*(14) = 0.8373, *p* = 0.4165, respectively. A paired-samples *t* test revealed no significant differences for test scenes presented concurrently with black letters (52.89%±1.33%) and novel white letters (53.13%±3.96%), *t*(14) = 0.1494, *p* = 0.8834, suggesting that the enhanced performance in prior experiments was not simply due to the perceptual novelty of the physical stimulus.

Together, these four experiments demonstrate that at behaviorally relevant points in time—operationally defined as a point of time that is important for the future execution or completion of an auditory or visual task—a memory trace of the visual field is automatically encoded into memory, enhancing later recognition of information even at unattended regions of visual space. This “screen capture” mechanism is likely to play an important role in the retrospective analysis of important events.

## Discussion

A defining characteristic of the human visual system is its ability to rapidly extract details of a scene, but it takes substantially longer to encode a scene into memory [1],[4]. However, recognition memory for scenes is remarkably good when given sufficient encoding time [23]. Traditionally, the encoding of pictures into memory has been studied using single-task, undivided attention paradigms exploring the effects of stimulus duration and visual and conceptual masking on effective encoding and later memory. Consequently, less is known about memory encoding under conditions of reduced attention.

What determines whether an item is remembered or forgotten? It has been shown that observers are very poor in discriminating or recognizing obvious and significant changes in scenes unless they happen to be attending to the item that was changed [24],[25]. As an extension of this, it is generally believed that focused spatial attention is necessary for a visual item to be encoded into memory [8]–[11]. In the present studies, however, even focal attention on the scenes in Experiment 1 was not sufficient to maintain familiar scenes in short-term memory.

In sharp contrast, in Experiment 2, when spatial attention was directed towards fixation on an attentionally demanding task, the presentation of a target item resulted in enhanced recognition memory for the scene presented concurrently with the target in the background. This result suggests a new mechanism that may play a role in determining what and when information about a scene is encoded into memory. A counterintuitive feature of this enhanced recognition memory effect is that it occurs in spite of the known effects of focusing of spatial attention around a target item [26],[27]. Our results indicate that target detection, engagement, or processing has a strong, non-stimulus-specific influence on memory formation—the enhanced encoding into memory of all items that are temporally coincident with a behaviorally relevant target event. The data suggest that behaviorally relevant points in time trigger a “temporal novelty” effect on memory encoding that appears to be a sufficient prerequisite for the successful encoding of visual stimuli into memory under conditions of reduced attention [28]–[30].

It is unlikely that this non-stimulus-specific influence on memory formation was due to the attentional blink [19],[31] suppressing scenes presented after focal targets were identified; indeed, recognition memory for scenes presented immediately before or after the temporal positions of the targets was still at chance. Moreover, the rate of presentation (two pictures/s) is considerably slower than rates that produce an attentional blink. In addition, recognition memory for the scenes presented before or after the temporal positions of the targets being at chance also suggests that the effects were not due to a general arousal [32] triggered by the onset of a perceptually novel stimulus and thereby increasing recognition memory for all subsequent scenes presented after the targets.

Perceptual learning for task-irrelevant peripheral stimuli can occur when attention is focused away from the peripheral stimuli and towards fixation and these learning effects are greatest for peripheral stimuli presented at the time of foveal target detection [33]–[35]. These results were surprising because it had generally been assumed that perceptual learning requires attention be focused on the target stimulus being learned. However, even in the absence of attention, it must be necessary for the target stimulus being learned to be encoded into memory for learning to occur. Here, we show that short-term memory for a peripheral scene is enhanced when it is presented at a behaviorally relevant point in time. It seems likely that a version of this “task-related screen capture” is one of the mechanisms that could support the phenomenon of perceptual learning in the absence of attention.

Recently, researchers have shown that repeated presentation of movie clips produces detectable “memory traces” in subsequent resting state activity in cat visual cortex [36]. It is plausible that given a behaviorally relevant point in time, a strong reverberation or memory trace was triggered and the residual of this imprint was being tapped into when performing the scene recognition task.

Finally, one might assume these results suggest that the processes associated with enhanced vividness, memory, and attention for novel events act globally throughout the visual field; however, Experiment 4 suggests that at first glance, perceptual novelty is not the source of these effects. When passively viewing the same displays as Experiment 2 and asked to perform the recognition memory task while ignoring the black distractor letters and novel white target letters, no significant differences were found in recognition performance. Overall, our results suggest a mechanism where traces of a visual scene are automatically encoded into memory at behaviorally relevant points in time regardless of the spatial focus of attention.

## Materials and Methods

All participants reported normal or corrected-to-normal visual acuity and gave informed consent to participate in this experiment, which was approved by the University of Washington Human Subjects Institutional Review Board. In every experiment prior to testing, participants performed a practice block of 24 trials. Each participant was then tested for a total of 240 trials, in 10 blocks of 24 trials. Blocks were separated by brief breaks.

Different participants participated in each of the five experiments. All received financial compensation in one 1 h session. Experiment 1 consisted of 12 participants (10 females, 2 males). Experiment 2 consisted of 11 participants (7 females, 4 males). Experiment 3 consisted of 11 participants (6 females, 5 males). Experiment 4 consisted of 15 participants (11 females, 4 males).

### Apparatus and Stimuli

Displays were presented on a 45 cm ViewSonic Graphics Series G90fB monitor at 1024×768 resolution, refreshed at 60 Hz. Participants sat with their eyes approximately 50 cm from the screen. The backgrounds of all displays were gray (15 cd/m^2^). Display items consisted of 192 700×700 pixel (28.07 degrees of visual angle) photographs depicting natural or urban scenes from eight distinct categories (i.e., mountains, cityscapes, etc). Scenes were obtained from the LabelMe Natural and Urban Scenes database [37] at 250×250 pixels of resolution, then up-sampled to 700×700 pixels of resolution.

Display items during the experiment were sampled from the 192 scenes with replacement. In each sequence, observers were shown 16 of these scenes at 133 ms per scene, followed by a blank ISI of 367 ms for a SOA of 500 ms.

### Scene Recognition Task

All experiments (1, 2, 3, and 4) used the scene recognition task. Following each rapid sequence of 16 full-field scenes, observers were presented with a test scene and asked to recall whether the test scene appeared in the previous RSVP sequence of scenes. The test scene was presented for 3,000 ms or until participants responded to whether they recognized the test scene from the RSVP stream with a “Y” or “N” on the keyboard. In 50% of the trials, the test scene was randomly drawn from the scenes presented in serial positions 9 to 16 of the RSVP; in the other 50% of trials, the test scene was drawn from the set of scenes not shown in the current RSVP stream. When test scenes were drawn from serial positions 9 to 16, there was a random 1/8 chance that the test scene matched the scene presented behind the white target letter in the RSVP stream, meaning that the white target letter task was irrelevant to the secondary recognition memory task and did not predict the test scene participants would be tested on. All scenes were sampled from our database with replacement. Distractors and target letters were embedded in randomly selected scenes over the entire session.

It is important to note that although our scene recognition task is similar to earlier studies that tested picture memory for novel scenes [1],[20], our task requires the participant to remember whether an already-familiar test picture appeared in the most recent sequence. Previous studies have used unfamiliar pictures on each trial. We presume that observers would have no difficulty detecting the presence or absence of a familiar scene in a sequence if they knew beforehand what scene to detect [3],[4]. In addition to the main result, the last scene in the RSVP sequence was often recognized with higher accuracy, in line with well-known recency effects of memory [38]–[43] and the fact that the last scene was not conceptually masked by a subsequent item. In Experiment 1, we only tested the second half of scenes presented in the RSVP to maintain consistency with subsequent experiments and therefore do not have data on potential performance differences for the first scene presented in the RSVP sequence. This new recognition memory task that measured participants' ability to encode a familiar set of scenes into short-term memory using RSVP sequences served as a starting point for examining potential temporally related enhancements to the encoding of briefly presented scenes into memory.

### Letter Target Identification Task

For the letter detection task (Experiment 2), a gray aperture (1 degree of visual angle) was embedded in the center of each scene and a random alphabetical letter (20 font size) was centered within the aperture. New random letters were embedded into the gray apertures of every scene, with the only requirement being that no duplicate letters could be presented within the same trial. Alphabetical letters were either black (indicating its identity as a distractor) or white (indicating its identity as a target; see Figure 1). In every trial, random black alphabetical letters representing distractors were embedded at central fixation in 15 of the scenes and a random white alphabetical letter representing the target was embedded in 1 scene. White target letters could only appear concurrently with scenes presented in serial positions 9 to 16 to avoid having white target letters presented at the onset of a RSVP stream. Participants were instructed to fixate on a point in the center of the screen and search for and identify a white target letter while memorizing the series of 16 scenes presented in RSVP.

In Experiment 2, immediately following the RSVP, participants were instructed to type the letter key corresponding to the identity of the white target letter for the current trial. Following the response to the letter detection task, participants performed the scene detection task. Participants were instructed to ignore the letter stream in Experiment 4.

### Auditory Target Identification Task

The auditory target detection task in Experiment 3 was similar to the letter detection task in Experiment 2 except the alphabetical letters were removed from the apertures centered in the scenes. Instead, an auditory tone was presented with each scene. Tones were sampled at 44,000 Hz, with durations of 50 ms. Baseline tones were presented at 261.50 Hz, while target tones were either 130.75 Hz or 523.0 Hz. Immediately following the RSVP stream, participants were instructed to discriminate the pitch of the unique tone as either lower or higher via key press, then were again presented with a test scene and asked to recall whether they recognized the scene from the RSVP stream.

## Abbreviations

RSVP: rapid serial visual presentation
